# Energy requirements for growth in the Yorkshire terrier

**DOI:** 10.1017/jns.2017.26

**Published:** 2017-05-24

**Authors:** Janet E. Alexander, Alison Colyer, Penelope J. Morris

**Affiliations:** WALTHAM Centre for Pet Nutrition, Waltham-on-the-Wolds, Melton Mowbray LE14 4RT, UK

**Keywords:** Energy requirements, Growth, Puppies, Yorkshire terriers, Dog breeds, NRC, National Research Council

## Abstract

The 2006 National Research Council (NRC) equation calculating puppy energy requirements does not account for reported breed differences in growth pattern. Energy requirements of toy breed puppies are unknown and it is unclear whether feeding guidelines should differ between breeds. Energy requirements of Yorkshire terrier (YT) puppies were observed over their first year of life and compared with those predicted by the NRC and those previously observed in large (Labrador retriever) and medium (miniature Schnauzer; MS) breed puppies. Twenty-two puppies (from eight litters) were offered complete and balanced diets to maintain ideal body condition score (BCS). Energy intake, body weight and BCS were recorded from 10 to 52 weeks of age. Every 12 weeks, health was monitored by veterinary examination, routine haematology and plasma biochemistry. Puppies remained clinically healthy with normal skeletal development throughout. After analysis by linear mixed models it was observed that the NRC equation overestimates YT energy requirements between 10 and 20 weeks of age by up to 324·3 (95 % CI 390·4, 258·2) kJ/kg^0·75^. Energy intake was lower (*P* < 0·05) in YT than Labradors until 29 weeks by up to 376·6 (95 % CI 477·4, 275·3) kJ/kg^0·75^ and lower than MS between 16 and 25 weeks by up to 216·3 (95 % CI 313·0, 119·7) kJ/kg^0·75^ (*P* < 0·05). Data indicate differences in toy, medium and large breed energy requirements for growth. The NRC equation for puppy energy requirements overestimated the requirements of this YT population, suggesting the need for breed-specific feeding guides for growth to avoid overfeeding.

Appropriate nutrition is a key factor for the optimal development of growing dogs. Over- or undernutrition during development can have lifelong effects on the health of dogs^(^^1^^,^^2^^)^; therefore it is therefore essential that appropriate food amounts are offered. The energy requirements of toy breed puppies have not been reported and it is unclear whether feeding guidelines for these dogs should differ from those determined for larger breeds. Breed-specific differences in growth patterns have been noted previously^(^^3^^–^^5^^)^ and therefore differences in energy requirements might be expected due to differences in adult body shape, size, temperament and coat type. Dobenecker *et al*.^(^^5^^)^ reported consistently higher energy intake in foxhound–boxer–Ingelheim Labrador mixed breed puppies than Beagle puppies up to 28 weeks of age. The authors suggest that during the major period of growth, energy requirement is not a function of age but of breed size. The 2006 National Research Council (NRC) equation for calculating the energy requirements of puppies was first suggested by Blanchard *et al.*^(^^6^^)^ and is in agreement with that suggested by Meyer & Zentek^(^^7^^)^. Neither equation, however, accounts for differences in breed. Brenten *et al.*^(^^8^^)^ investigated the energy requirements of miniature Schnauzer and Labrador retriever puppies and observed that for miniature Schnauzer puppies, the NRC equation initially overestimates and subsequently underestimates energy requirements while those of Labrador retrievers were met. As accurate feeding guides are vital for healthy growth in puppies, the primary objective of this study was to investigate the energy requirements of a toy breed, namely the Yorkshire terrier, from 10 weeks to 1 year of age. A secondary objective was to compare the observed energy requirements of Yorkshire terrier puppies over this time with those of medium and large breeds raised under the same conditions.

## Experimental design

This work was approved by the WALTHAM Animal Welfare and Ethical Review Body and conducted under the authority of the Animals (Scientific Procedures) Act 1986. A total of twenty-two puppies from eight litters took part in the study. Puppies were housed with their mother until weaning at 8 weeks of age, in litter groups until 10 weeks of age, and in pairs thereafter. In all cases housing consisted of environmentally enriched kennels with constant access to an outdoor area. All puppies received socialisation and training sessions daily and access to large outdoor play areas.

Puppies were offered either a commercially available wet (Cesar^®^ Puppy Chicken and Rice; Mars Petcare) or a dry (Royal Canin^®^ Yorkshire Terrier Junior; Mars Petcare) diet or both in a mixed feeding regimen. Diet was randomly allocated within each litter. Between the ages of 10 and 26 weeks, puppies were offered their daily ration in 3 × 30 min meals and in 2 × 30 min meals from 27 to 52 weeks of age. Free access to drinking water was given at all times. Diets underwent nutritional analysis (Eurofins) and the results used to calculate the predicted metabolisable energy content of the diets according to the equation described in NRC (2006)^(^^9^^)^ which in turn was used to calculate the energy intake of the dogs. Food intake was recorded immediately following each meal as the mass of food offered minus the mass of food returned (Sartorius UK Ltd). Feeding allowances were calculated from amounts consumed during the previous week and adjusted weekly with the aim of maintaining puppies at an ideal body condition score throughout the study. Although not fully validated in puppies, body condition score was evaluated weekly using the WALTHAM S.H.A.P.E (Size, Health and Physical Evaluation) guide^(^^10^^)^, which uses visual and palpable characteristics to determine the amount of subcutaneous and abdominal fat. Each category is assigned an alphabetical character from A (underweight) to G (obese), with D representing ideal^(^^10^^)^. Body weight was recorded weekly using calibrated scales (Sartorius UK Ltd).

Puppies underwent a physical examination at the start and end of the study. At 3, 6, 9 and 12 months of age, a fasted (>12 h) jugular blood sample was collected. Lithium–heparin anticoagulated blood was used for the determination of standard biochemistry parameters; total protein, albumin, phosphate, alkaline phosphatase (ALP), alanine transaminase (ALT), aspartate aminotransferase (AST), Ca, cholesterol, urea, creatinine, TAG and glucose using an AU400 (Olympus) analyser. EDTA anticoagulated blood was collected for the measurement of standard haematology parameters; leucocyte and erythrocyte counts, Hb concentration, packed cell volume percentage, platelet count, mean corpuscular volume, mean corpuscular Hb, and number and percentage of lymphocytes, monocytes and granulocytes were analysed via a Mythic cell counter (Orphée SA).

Predicted maintenance energy requirements were calculated, using the NRC (2006)^(^^9^^)^ puppy energy requirement equation assuming adult body weight (kg) was that measured at 52 weeks of age.

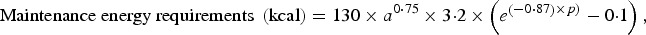

where *a* = body weight observed (kg), *p* = (body weight observed/body weight at 52 weeks), and *e* = base natural log (2·718). (To convert requirements in kcal to kJ, multiply by 4·184.)

Once calculated, actual energy intake of the dogs was compared with the energy requirements predicted by the NRC 2006 equation^(^^9^^)^. A linear mixed-effects model was fitted to the observed weekly average daily intakes (kJ/kg^0·75^), the NRC predicted maintenance energy requirement (kJ/kg^0·75^) and the difference between the observed and NRC predicted energy requirement. For each model, dog nested in litter was used as the random effect with an autoregressive correlation structure, of order 1, to take account of the correlation between successive measurements within a dog. The calculated energy requirements of the Yorkshire terriers were then compared with those of Labradors and miniature Schnauzers as previously described^(^^8^^)^. Breed, week and their interactions were fitted as categorical fixed effects. Comparisons between breeds were performed for each week using a family-wise error rate of 5 %. Linear mixed-model analysis was carried out using the *nlme* and *multcomp* packages of R v2.15.0 statistical software^(^^11^^)^. Means and contrasts are reported with 95 % family-wise intervals.

## Results

All dogs remained healthy during the course of the study with no skeletal abnormalities as judged by clinical veterinary examination at 52 weeks. Haematological (data not shown) and biochemical parameters were within normal ranges, significant (*P* < 0·05) between-breed differences were observed in a number of biochemical parameters over the first year (Supplementary Table S1). All dogs maintained ideal body condition score throughout the study (data not shown). As expected, body weight increased significantly with time.

**Fig. 1.**
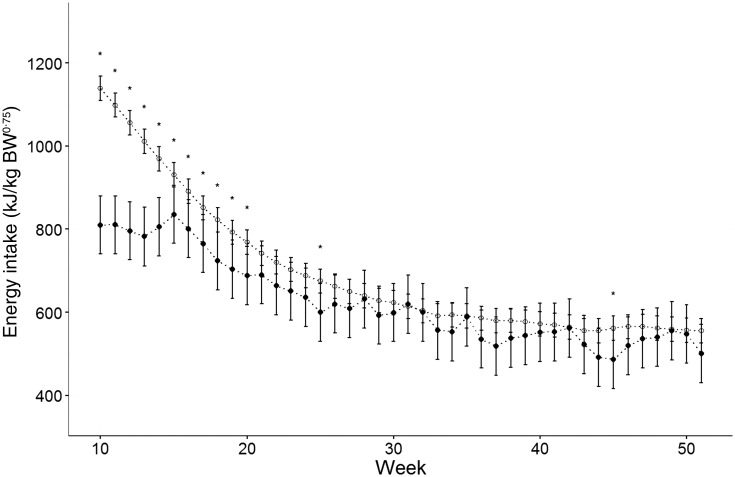
Actual energy intake of twenty-two Yorkshire terriers (●) in comparison with the energy requirements predicted by the National Research Council (2006) equation^(^^9^^)^ (○). Data are means, with 95 % confidence intervals represented by vertical bars. * Significant difference (*P* < 0·05).

**Fig. 2.**
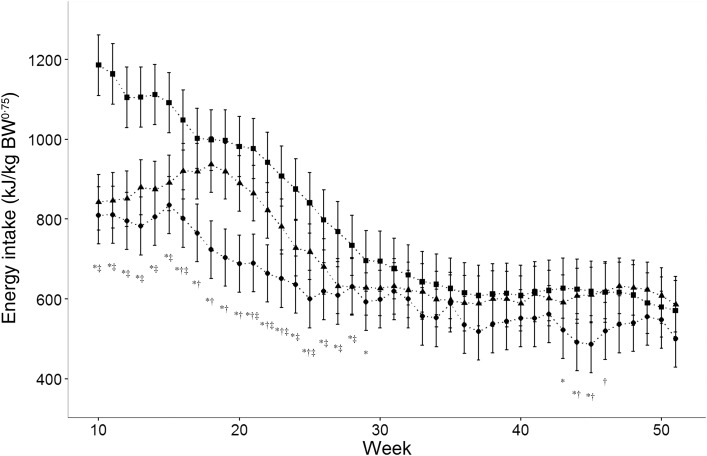
Actual energy intake of twenty-two Yorkshire terriers (YT; ●) in comparison with those reported for Labrador retrievers (LAB; ■) and miniature Schnauzers (MS; ▲)^(^^8^^)^. Data are means, with 95 % confidence intervals represented by vertical bars. * Significant difference between YT and LAB (*P* < 0·05). † Significant difference between YT and MS (*P* < 0·05). ‡ Significant difference between LAB and MS (*P* < 0·05).

The energy required to maintain optimal body condition significantly decreased from a mean of 810 (95 % CI 740, 880) kJ/kg^0·75^ per d at 10 weeks of age to 500 (95 % CI 431, 570) kJ/kg^0·75^ per d at 52 weeks of age (Fig. 1). The energy intake per kg^0·75^ (Fig. 1) was overestimated by the NRC (2006)^(^^9^^)^ equation from 10 to 20 weeks of age whilst the values were comparable from 21 to 52 weeks of age with the exception of weeks 25 and 45. When compared with the energy requirement values reported by Brenten *et al.*^(^^8^^)^ (Fig. 2), there was a significant interaction of breed with time (*P* < 0·0001) with the energy intake of Yorkshire terriers being significantly less than that of Labrador retrievers from 10 to 29 weeks of age (*P* ≤ 0·05) by 77·4 (95 % CI 132·6, 61·1) to 376·6 (95 % CI 477·4, 275·3) kJ/kg^0·75^. No difference was observed in energy requirements between miniature Schnauzers and Yorkshire terriers from week 10 to week 15 (*P* > 0·05). However, from week 16 to week 23 and in week 25 the energy requirements of Yorkshire terriers were significantly lower (*P* < 0·01) by 118·8 (95 % CI 215·5, 5·2) kJ/kg^0·75^ to 21·76 (95 % CI 313·0, 119·7) kJ/kg^0·75^. There was little effect of breed after 29 weeks of age.

## Discussion

The data presented here indicate that there are breed-related differences in energy requirements for growth up to 29 weeks of age and that the NRC equation predicating energy requirements overestimates for Yorkshire terriers from 10 to 20 weeks of age.

The provision of accurate feeding guides is essential if under- and overfeeding are to be prevented. Small and toy breeds have been identified as being especially prone to becoming overweight which has in turn been shown to lead to reduced life expectancy and a number of pathological conditions^(^^12^^)^. The causes of excess body weight are multifactorial, but healthy growth in puppyhood plays a key part in maintaining a healthy adult body weight^(^^12^^,^^13^^)^.

When energy intake was determined over the first year of life in the Yorkshire terriers, as expected, it reduced with age as growth slowed and the dogs approached their adult size. At 1 year of age the mean energy intake for the Yorkshire terriers in this study (500·4, 95 % CI 430·5, 570·3 kJ/kg^0·75^ per d), was comparable with that reported previously^(^^14^^)^ for adult Yorkshire terriers aged between 16 and 52 months (472·8, 95 % CI 284·5–765·7 kJ/kg^0·75^ per d), indicating that the Yorkshire terriers in the present study had energy requirements normal for their breed under kennel conditions. When the energy requirements of the Yorkshire terriers, Labrador retrievers and miniature Schnauzers were compared, breed-specific patterns were observed. In agreement with data previously reported for miniature Schnauzer puppies^(^^8^^)^, Yorkshire terrier puppies required lower energy intake to maintain ideal body condition score than did Labrador retriever puppies until 23 weeks of age. The energy intakes of the miniature Schnauzer puppies were very similar to those of the Yorkshire terriers until 16 weeks of age when they diverged, possibly due to a longer rapid growth phase in the miniature Schnauzers. Such breed-specific variance in growth pattern has been noted previously^(^^3^^–^^5^^)^ and might be expected due to differences in adult body weight, size, composition, temperament and coat type, all of which have to affect energy requirements. Although in the present study body composition was not determined, breed differences in lean and fat mass have previously been reported which could affect energy requirements during growth^(^^15^^)^. Little difference in biochemical markers of energy metabolism were observed, no significant between-breed differences were determined in the levels of amino acid-converting enzymes alanine transaminase (ALT) and aspartate aminotransferase (AST), although TAG concentrations (indicative of the level of hepatic resynthesis) were significantly different between 10 and 14 weeks. Levels of urea, creatinine and hence their ratio were significantly different between Yorkshire terriers and Labrador retrievers from 34 weeks, possibly indicating differences in levels of protein metabolism. However, this difference was only apparent during the later phase of growth when the energy requirements were similar between breeds. The data presented here support the hypothesis that during the major period of growth, energy requirement is closely associated with breed size as well as age. Collectively the data suggest that breed differences in energy requirements should be taken into account when recommending feeding amounts during growth; however, present feeding guides are based on ‘typical’ growth data that use only a single equation (NRC, 2006^(^^9^^)^).

The NRC 2006 equation calculating the energy requirements of puppies was developed from equations^(^^6^^,^^7^^)^ which do not take breed differences into account. Previously, this equation was observed to overestimate for miniature Schnauzers up to 15 weeks of age, while the requirements of Labrador retrievers were reported to be as predicted to 16 weeks^(^^8^^)^. In the present study, an overestimation of energy requirement up to 21 weeks of age was observed when the actual energy intake of the Yorkshire terriers was compared with those predicted by the NRC equation^(^^9^^)^. From 21 weeks the predicted energy requirements were not significantly different from the observed energy intake with the exception of weeks 25 and 45. While week 25 corresponded with the age of neutering for most dogs and this procedure may have affected energy intake, no explanation for the difference at 45 weeks is apparent. If used as the sole guideline for feeding the Yorkshire terrier puppies, the NRC equation could have resulted in excess energy intake and hence excess body weight. One explanation for the overestimation of energy requirement could be the level of physical activity of the Yorkshire terrier and miniature Schnauzer puppies in these studies. If activity was lower than expected in this population, energy requirements would be lower than predicted. Differences in energy intake between pet and kennelled puppies have previously been attributed to differences in physical activity levels^(^^5^^,^^16^^)^. However, the dogs in the present study were housed in surroundings as similar to a domestic household as can be achieved in a kennel environment and had a standardised care package including exercise and play sessions. Although differences in activity level in kennelled dogs may affect energy requirements compared with the pet population, this is unlikely to influence the between-breed differences in growth pattern observed here as all the dogs were kennelled under the same conditions. In future studies accelerometry could be used to compare the physical activity levels of puppies of different breeds.

### Conclusion

This study illustrates differences in growth pattern between toy, medium and large breeds, in terms of energy requirements. The data indicate that one general equation may be unsuitable for the calculation of energy requirements for growth in puppies of differing breeds and support the need for further research into the need for breed-specific feeding guides.

## Supplementary material

For supplementary material accompanying this paper visit http://dx.doi.org/10.1017/jns.2017.26. click here to view supplementary material

## References

[1] Hedhammar A, Wu F, Krook L, (1974) Overnutrition and skeletal disease: an experimental study in growing great Dane dogs. Cornell Vet 64, Suppl. 5, 5–160. 4826273

[2] Meyer H & Zentek J (1992) Über den Einfluß einer unterschiedlichen Energieversorgung wachsender Doggen auf Körpermasse und Skelettentwicklung. 1. Mitteilung: Körpermasseentwicklung und Energiebedarf (Influence of various levels of energy intake on development of body weight and skeleton in growing great Danes 1. Growth rate and energy requirement). J Vet Med Ser A 39, 130–141. 1590036

[3] Kienzle E & Rainbird A (1991) Maintenance energy requirement of dogs: what is the correct value for the calculation of metabolic body weight in dogs? J Nutr 121, Suppl. 11, S39–S40. 1941233 10.1093/jn/121.suppl_11.S39

[4] Hawthorne AJ, Booles D, Nugent PA, (2004) Body-weight changes during growth in puppies of different breeds. J Nutr 134, Suppl. 8, 2027S–2030S. 15284394 10.1093/jn/134.8.2027S

[5] Dobenecker B, Endres V & Kienzle E (2013) Energy requirements of puppies of two different breeds for ideal growth from weaning to 28 weeks of age. J Anim Physiol Anim Nutr 97, 190–196. 10.1111/j.1439-0396.2011.01257.x 22106988

[6] Blanchard G, Grandjean D & Paragon B-M (1998) Calculation of a dietary plan for puppies. J Anim Physiol Anim Nutr 80, 54–59.

[7] Meyer H & Zentek J (1998) Ernährung des Hundes (Nutrition of the Dog), 3rd ed. Stuttgart: Verlag Eugen Ulmer.

[8] Brenten T, Morris PJ, Salt C, (2014) Energy intake, growth rate and body composition of young Labrador retrievers and miniature Schnauzers fed different dietary levels of vitamin A. Br J Nutr 111, 2104–2111. 24666690 10.1017/S0007114514000543

[9] National Research Council (2006) Energy requirements of dogs In Nutrient Requirements of Dogs and Cats, pp. 38–39. Washington, DC: National Academies Press.

[10] German AJ, Holden SL, Moxham GL, (2006) A simple, reliable tool for owners to assess the body condition of their dog or cat. J Nutr 136, 2031S–2033S. 16772488 10.1093/jn/136.7.2031S

[11] R Development Core Team (2012) R: A Language and Environment for Statistical Computing. Vienna, Austria: R Foundation for Statistical Computing http://cran.r-project.org

[12] German AJ (2006) The growing problem of obesity in dogs and cats. J Nutr 136, 1940S–1946S. 16772464 10.1093/jn/136.7.1940S

[13] Courcier E, Thomson R, Mellor D, (2010) An epidemiological study of environmental factors associated with canine obesity. J *Small Anim Pract* 51, 362–367. 20402841 10.1111/j.1748-5827.2010.00933.x

[14] Serisier S, Weber M, Feugier A, (2013) Maintenance energy requirements in miniature colony dogs. J Anim Physiol Anim Nutr 97, 60–67. 10.1111/jpn.12044 23639018

[15] Speakman J, Van Acker A & Harper E (2003) Age-related changes in the metabolism and body composition of three dog breeds and their relationship to life expectancy. Aging Cell 2, 265–275. 14570234 10.1046/j.1474-9728.2003.00061.x

[16] Liesegang A, Fuglistaller C & Wichert B (2009) Puppy feeding in Switzerland. Schweiz Arch Tierheilkd 51, 521–528. 10.1024/0036-7281.151.11.521 19885797

