# Supplementary material for: Energy requirements for growth in the Yorkshire terrier
Source: J Nutr Sci. 2017 May 24;6:e26. doi: 10.1017/jns.2017.26 (PMC5468749; doi:10.1017/jns.2017.26)
Supplement: Supplementary file 1 [file S204867901700026Xsup001.docx]

**Supplementary Table S1.** Biochemical parameters

|  |  | Yorkshire terrier | Labrador |  |
| --- | --- | --- | --- | --- |
| Parameter | Age group (week) | Mean (95% CI) | Mean (95% CI) | *P* |
| Total protein (g/L) | 10-14 | 53.5 (51.9, 55.1) | 49.5 (47.9, 51.1) | <0.001* |
|  | 14-18 | 52.8 (50.3, 55.3) | 51.2 (49.7, 52.7) | 0.693 |
|  | 22-26 | 55.4 (53.1, 57.1) | 53.1 (51.6, 54.7) | 0.056 |
|  | 34-38 | 56.4 (54.8, 57.9) | 53.0 (51.4, 54.6) | <0.001* |
|  | 50-54 | 57.5 (55.7, 59.3) | 55.4 (53.7, 57.1) | 0.192 |
| Albumin (g/L) | 10-14 | 27.3 (26., 28.54) | 25.15 (23.7, 26.6) | 0.036* |
|  | 14-18 | 27.4 (25.6, 29.3) | 26.89 (25.5, 28.3) | 0.998 |
|  | 22-26 | 29.0 (27.7, 30.3) | 28.28 (26.9, 29.7) | 0.948 |
|  | 34-38 | 29.6 (28.4, 30.9) | 27.55 (26.1, 29.0) | 0.038* |
|  | 50-54 | 30.7 (29.3, 32.1) | 29.08 (27.6, 30.6) | 0.300 |
| Phosphate (mmol/L) | 10-14 | 2.4 (2.3, 2.6) | 2.8 (2.7, 3.0) | <0.001* |
|  | 14-18 | 2.4 (2.2, 2.6) | 2.8 (2.7, 3.0) | <0.001* |
|  | 22-26 | 1.9 (1.7, 2.0) | 2.6 (2.5, 2.8) | <0.001* |
|  | 34-38 | 1.5 (1.4, 1.7) | 2.2 (2.0, 2.4) | <0.001* |
|  | 50-54 | 1.4 (1.2, 1.5) | 1.7 (1.5, 1.9) | 0.002* |
| ALT (U/L) | 10-14 | 24.1 (21.0, 27.7) | 21.0 (18.7, 23.4) | 0.258 |
|  | 14-18 | 20.6 (16.4, 26.0) | 23.0 (20.9, 25.4) | 0.879 |
|  | 22-26 | 27.2 (23.7, 31.3) | 29.8 (26.9, 32.9) | 0.762 |
|  | 34-38 | 30.5 (26.8, 34.9) | 34.0 (30.3, 38.0) | 0.589 |
|  | 50-54 | 31.5 (26.9, 37.0) | 34.7 (30.5, 39.5) | 0.860 |
| AST (U/L) | 10-14 | 25.8 (22.7, 29.3) | 27.8 (24.3, 31.9) | 0.931 |
|  | 14-18 | 30.7 (25.3, 37.2) | 30.2 (26.4, 34.4) | 1.000 |
|  | 22-26 | 26.7 (23.4, 30.4) | 28.4 (24.9, 32.5) | 0.976 |
|  | 34-38 | 30.2 (26.7, 34.3) | 31.2 (27.1, 35.6) | 1.000 |
|  | 50-54 | 28.2 (24.5, 32.5) | 31.2 (26.9, 35.9) | 0.856 |
| Calcium (μmol/L) | 10-14 | 2.9 (2.8, 3.0) | 2.8 (2.7, 2.9) | 0.253 |
|  | 14-18 | 2.8 (2.7, 2.9) | 2.8 (2.7, 2.9) | 1.000 |
|  | 22-26 | 2.7 (2.6, 2.8) | 2.8 (2.7, 2.9) | 0.616 |
|  | 34-38 | 2.6 (2.6, 2.7) | 2.5 (2.4, 2.6) | 0.426 |
|  | 50-54 | 2.6 (2.5, 2.7) | 2.6 (2.5, 2.6) | 1.000 |
| Cholesterol (mmol/L) | 10-14 | 5.4 (4.5, 6.4) | 6.0 (4.7, 7.3) | 0.976 |
|  | 14-18 | 6.5 (5.4, 7.6) | 6.7 (5.4, 8.0) | 1.000 |
|  | 22-26 | 5.5 (4.6, 6.5) | 7.1 (5.8, 8.3) | 0.140 |
|  | 34-38 | 5.2 (4.3, 6.2) | 6.3 (5.0, 7.6) | 0.538 |
|  | 50-54 | 5.6 (4.6, 6.6) | 6.6 (5.3, 7.9) | 0.684 |
| Urea (mmol/L) | 10-14 | 3.3 (2.4, 4.1) | 3.3 (2.5, 4.2) | 1.000 |
|  | 14-18 | 3.6 (2.3, 5.0) | 4.9 (4.1, 5.7) | 0.271 |
|  | 22-26 | 4.6 (3.7, 5.5) | 4.8 (4.0, 5.6) | 1.000 |
|  | 34-38 | 5.2 (4.3, 6.0) | 5.5 (4.7, 6.4) | 0.988 |
|  | 50-54 | 5.5 (4.5, 6.4) | 7.1 (6.2, 8.1) | 0.009* |
| Creatine (mmol/L) | 10-14 | 41.7 (38.2, 45.5) | 41.9 (37.5, 46.8) | 1.000 |
|  | 14-18 | 42.8 (38.4, 47.8) | 46.8 (42.0, 52.2) | 0.711 |
|  | 22-26 | 59.9 (54.8, 65.4) | 67.9 (60.9, 75.8) | 0.185 |
|  | 34-38 | 68.0 (62.4, 74.1) | 86.0 (77.0, 96.0) | <0.001* |
|  | 50-54 | 70.9 (64.7, 77.7) | 86.1 (76.9, 96.3) | 0.006* |
| Triglycerides (mmol/L) | 10-14 | 0.6 (0.5, 0.7) | 0.4 (0.3, 0.5) | <0.001* |
|  | 14-18 | 0.5 (0.4, 0.6) | 0.4 (0.4, 0.5) | 0.571 |
|  | 22-26 | 0.5 (0.4, 0.6) | 0.5 (0.4, 0.5) | 0.673 |
|  | 34-38 | 0.5 (0.4, 0.6) | 0.4 (0.3, 0.5) | 0.060 |
|  | 50-54 | 0.4 (0.4, 0.5) | 0.4 (0.3, 0.5) | 0.924 |
| ALP (U/L) | 10-14 | 134.7 (116.3, 156.1) | 129.5 (108.6, 154.5) | 1.000 |
|  | 14-18 | 102.0 (84.3, 123.3) | 120.8 (101.5, 143.7) | 0.548 |
|  | 22-26 | 88.8 (76.6, 103.1) | 104.6 (87.9, 124.6) | 0.443 |
|  | 34-38 | 61.6 (53.2, 71.2) | 68.2 (57.2, 81.9) | 0.897 |
|  | 50-54 | 37.4 (32.0, 43.6) | 46.5 (38.9, 55.7) | 0.152 |
| Glucose (mmol/L) | 10-14 | 6.5 (6.2, 6.8) | 6.5 (6.1, 6.8) | 1.000 |
|  | 14-18 | 6.6 (6.1, 7.0) | 6.3 (5. 9, 6.6) | 0.761 |
|  | 22-26 | 6.3 (5.9, 6.6) | 6.0 (5.7, 6.4) | 0.895 |
|  | 34-38 | 5.9 (5.6, 6.2) | 5.5 (5.1, 5.9) | 0.160 |
|  | 50-54 | 6.0 (5.6, 6.3) | 5.5 (5.1, 5.8) | 0.090 |

ALT: alanine aminotransferase, AST: aspartate aminotransferase, ALP: Alkaline phosphatase.

Asterisk indicates a significant difference between breeds, *P*<0.05.
